# Risk of Fractures and Falls during and after 5-α Reductase Inhibitor Use: A Nationwide Cohort Study

**DOI:** 10.1371/journal.pone.0140598

**Published:** 2015-10-15

**Authors:** David Robinson, Hans Garmo, Pär Stattin, Karl Michaëlsson

**Affiliations:** 1 Department of Surgery and Perioperative Sciences, Urology and Andrology, Umeå University, Umeå, Sweden; 2 Department of Urology, Ryhov County Hospital, Jönköping, Sweden; 3 Regional Cancer Centre, Uppsala University Hospital, Uppsala, Sweden; 4 King´s College London, Medical School, Division of Cancer Studies, Cancer Epidemiology Group, London, United Kingdom; 5 Orthopedics Section, Department of Surgical Sciences and Uppsala Clinical Research Center, Uppsala University, Uppsala, Sweden; Cedars-Sinai Medical Center, UNITED STATES

## Abstract

**Background:**

Lower urinary tract symptoms are common among older men and 5-α reductase inhibitors **(**5-ARI) are a group of drugs recommended in treating these symptoms. The effect on prostate volume is mediated by a reduction in dihydrotestosterone; however, this reduction is counterbalanced by a 25% rise in serum testosterone levels. Therefore, 5-ARI use might have systemic effects and differentially affect bone mineral density, muscular mass and strength, as well as falls, all of which are major determinants of fractures in older men.

**Methods:**

We conducted a nationwide cohort study of all Swedish men who used 5-ARI by comparing their risk of hip fracture, any type of fracture and of falls with matched control men randomly selected from the population and unexposed to 5-ARI.

**Results:**

During 1 417 673 person-years of follow-up, 10 418 men had a hip fracture, 19 570 any type of fracture and 46 755 a fall requiring hospital care. Compared with unexposed men, current users of 5-ARI had an adjusted hazard ratio (HR) of 0.96 (95% CI 0.91–1.02) for hip fracture, an HR of 0.94 (95% CI 0.90–0.98) for all fracture and an HR of 0.99 (95% CI 0.96–1.02) for falls. Former users had an increased risk of hip fractures (HR 1.10, 95% CI 1.01–1.19).

**Conclusion:**

5-ARI is safe from a bone health perspective with an unaltered risk of fractures and falls during periods of use. After discontinuation of 5-ARI, there is a modest increase in the rate of fractures and falls.

## Introduction

Lower urinary tract symptoms (LUTS) are common among older men [1, 2]. Several guidelines recommend 5-α reductase inhibitors **(**5-ARIs) as a first treatment choice for LUTS in men with an enlarged prostate [3–5] since 5-ARIs reduce the volume of the prostate and thereby the risk of urinary retention and surgery [6–9]. The effect on prostate volume is mediated by a local pronounced reduction in dihydrotestosterone (DHT), which is the primary active androgen in the prostate [9, 10]. There is also a decrease in circulating DHT. However, this reduction is counteracted by a 25% rise in serum testosterone [9], which, by aromatization, might lead to higher circulating serum estradiol [11, 12]. Higher levels of circulating androgen and estrogen are positively associated with greater muscle and bone mass, improved postural balance and lower fracture risk in men [13–16]. In accordance, the risks of osteoporosis [17] and sarcopenia [18], low physical performance [19, 20], fall risk [19, 20], and fractures in some [13, 21], but not in all studies [22], are higher in men with low serum testosterone levels. Therefore, 5-ARI use might have systemic effects and differentially affect bone mineral density (BMD), muscular mass and strength, as well as falls, all of which are major determinants of fractures in older men [13, 19, 23–28]. These older men are especially vulnerable to consequent comorbidity and sustained high mortality after the fragility fracture event[29]. There are two clinically approved 5-ARIs on the pharmaceutical market: finasteride and dutasteride. The latter compound has a more complete suppression of the 5α-reductase isoenzymes and this treatment therefore leads to a more pronounced decrease in serum DHT concentrations [9, 30].

The potential net effect of 5-ARI treatment on BMD is at present unclear, with biological plausibility in either direction possible [17, 31]. Two small randomised clinical trials showed no certain effect by finasteride on BMD [32, 33].

Three previous attempts have sought to evaluate the relation between 5-ARI use and fracture risk by observational study designs. The approximately 20% relative reduction in risk of fracture observed with use of 5-ARI in one previous case-control study from the USA [34] was not confirmed in a Danish case-control study [35], or in a case-control study from the UK [36]. Importantly, given that elderly men are especially vulnerable to the often devastating consequences of fragility fractures, including high mortality rates [37–40], further analyses are warranted. None of the previous studies have evaluated rates of fracture with a cohort design or evaluated risk of falling, an important determinant of fracture risk, after initiation of 5-ARI. Any association between 5-ARI and fracture risk is probably modest and thus large studies are needed to establish sufficient power. We therefore conducted a nationwide cohort study of all Swedish men who used 5-ARI treatment and compared their future rate of hip fracture, any type of fracture and of falls with rates in men randomly selected from the population and unexposed to 5-ARI.

## Methods

### Study design

Data on exposure to finasteride or dutasteride, the two 5-ARIs currently in existence, were retrieved from The Swedish Prescribed Drug Register, which includes all prescriptions dispensed in Sweden since July 2005[41]. This register contains information on the amount and dose for each filled drug prescription as well as date of prescription and dispensing. All men older than 30 years who were exposed to 5-ARI at any time between 1 January 2006 to 31 December 2008 were included. In the cohort we additionally included from the Swedish Population Register randomly selected but frequency-matched men by year of birth and county of residence in a ratio of 5:1. These men were unexposed to previous use of 5-ARI and α-blockers and had not undergone a transurethral resection of their prostate (TUR-P; see below). Using the unique personal identity number assigned to each Swedish resident, the exposed and unexposed men were linked to a number of other nationwide health care registers and demographic databases [42, 43]. From 1987, the National Patient Register holds complete information, coded according to International Classification of Diseases (ICD-9 or ICD-10), on in-patient care and since 2001 information on out-patient hospital care. We identified such diagnoses and surgical procedures as TUR-P (KED 00–98) and surgical castrations (KFC 10, 15). From this register, we additionally identified outcome diagnoses: hip fracture ICD10 codes (S720-S722), any fracture (codes S12-S92) and any type of accidental fall (codesW00-W19). Men with a hip fracture diagnosis before 31 December 2005 were excluded. Endocrine treatment against prostate cancer and use of α-blockers was also identified from The Swedish Prescribed Drug Register. Because of convincing evidence connecting the metabolic syndrome to an increased risk not only to benign prostatic hyperplasia (BPH)/LUTS but also to poor physical performance and higher risk of fracture, we identified from the prescription register usage of medications related to the metabolic syndrome, such as statins and anti-hypertensive drugs [44–46]. We considered individual differences in comorbidities at baseline, identified from ICD codes from the National Patient Register, by classifying exposed and unexposed men into four categories of the Charlson weighted comorbidity index (CCI)[47]. The Longitudinal Integration Database for Health Insurance and Labour Market Studies (LISA) is a nationwide demographic database with data on socioeconomic factors[48]. From this database, we obtained information on educational level, which was categorised as low (≤9 years of school), medium (10–12 years) and high (≥13 years), which in Sweden corresponds to mandatory school, high school and college or university. The study was approved by the Research Ethics Board at Umeå University Hospital.

### Statistics

We estimated age- and multivariate-adjusted hazard ratios (HRs) for risk of hip fracture, any fracture and falls by Cox proportional hazards regression using age as a time scale and with time-updated information on exposures and covariates. All men were followed from 1 January 2006 until date of event, date of death, emigration, start of endocrine treatment against prostate cancer or to the end of the observation period, i.e. 31 December 2011, whichever occurred first. Exposure of 5-ARI was first categorised into current and former use based on dates of first prescription and last prescription with duration of use defined by daily doses (DDD). Current users were analysed in categories (<1, 1–2, 2–3, 3–4 and >4 years). Former users were separated into duration of use and time since last use by four categories: usage within the last year for a total duration of <1 year, usage within the last year for a total duration of ≥1 year, usage more than 1 year ago with a total duration of use of <1 year and usage more than 1 year ago with a total duration of use for ≥1 year. In Sweden, the maximum dispensed doses at the pharmacy are 100 days. We monitored any discontinuation stepwise [49]. If no new prescription were collected at the pharmacy 90 days after the previous doses would have been consumed, we regarded this individual as a former user from that point and onwards. If there were any new prescription collected at the pharmacy after this period, he was again regarded as a current user.

To minimise potential bias the directed acyclic graph approach was used to identify a suitable multivariable model [50, 51]. Exposure to calcium antagonists, angiotensin converting enzyme inhibitors, angiotensin II inhibitors, β-blockers, statins, oral diabetes drugs, insulin and diuretics (subdivided into potassium-sparing, loop, thiazide and miscellaneous diuretics) were included in the models as separate binary time-dependent covariates (never user or ever user, defined as at least one prescription in the The Swedish Prescribed Drug Register). Additionally included in the multivariable model were number of Charlson comorbidities (0, 1, 2, 3+) and education (high, medium, low). We also adjusted for time-dependent alpha-blocker use (ever, never) and TUR-P. Finally, we adjusted for previous fractures (0–1 and 1–5 years before start of follow-up) and previous hospitalisation due to fall (0–1 and 1–5 years before start of follow-up).

In a first sensitivity analysis we separately considered exposure to finasteride and dutasteride. Second, for a more accurate classification of duration of 5-ARI use, we restricted the analysis to men with their first exposure after 31 December 2005. If there were no prescription collected between 1 July to 31 December 2005, we regarded the individual as previously unexposed in that the drugs are normally dispensed every third month. If there were a prescription within this time frame, the individual was excluded from this specific sensitivity analysis.

## Results

We included 48 041 men exposed to 5-ARI and 219 113 men not exposed to 5-ARI, with a combined mean age of 72 years. 5-ARI users had slightly more comorbidities and medication use associated with the metabolic syndrome Table 1.

**Table 1:** Baseline characteristics for men exposed to 5 alpha reductase inhibitors (5-ARI) and selected non exposed men.

	5-ARI (n = 48 041)		Non exposed (n = 219 113)		All (n = 267 154)	
**Age, Mean (SD)**	72.1	(11.0)	72.4	(11.1)	72.4	(11.1)
**Education level, n (%)**						
High	10546	(22.0)	39664	(18.1)	50210	(18.8)
Middle	17394	(36.2)	76511	(34.9)	93905	(35.2)
Low/Missing	20101	(41.8)	102938	(47.0)	123039	(46.1)
**CCI, n (%)**						
0	28398	(59.1)	136954	(62.5)	165352	(61.9)
1	9416	(19.6)	39791	(18.2)	49207	(18.4)
2	5387	(11.2)	22828	(10.4)	28215	(10.6)
3+	4840	(10.1)	19540	(8.9)	24380	(9.1)
**Calcium Antagonist, n (%)**						
No	39425	(82.1)	187810	(85.7)	227235	(85.1)
Yes	8616	(17.9)	31303	(14.3)	39919	(14.9)
**ACE inhibitors, n (%)**						
No	39039	(81.3)	183641	(83.8)	222680	(83.4)
Yes	9002	(18.7)	35472	(16.2)	44474	(16.6)
**ANGII, n (%)**						
No	42015	(87.5)	198889	(90.8)	240904	(90.2)
Yes	6026	(12.5)	20224	(9.2)	26250	(9.8)
**Statins, n (%)**						
No	35453	(73.8)	173554	(79.2)	209007	(78.2)
Yes	12588	(26.2)	45559	(20.8)	58147	(21.8)
**Diabetes, n (%)**						
No diabetes	42743	(89.0)	196586	(89.7)	239329	(89.6)
Insulin	1548	(3.2)	7348	(3.4)	8896	(3.3)
Tablets	3750	(7.8)	15179	(6.9)	18929	(7.1)
**Diuretics, n (%)**						
No diuretics	36289	(75.5)	171115	(78.1)	207404	(77.6)
Potassium-sparing- diuretics	1131	(2.4)	4553	(2.1)	5684	(2.1)
Combined diuretics	1818	(3.8)	7470	(3.4)	9288	(3.5)
Loop diuretics	6237	(13.0)	25536	(11.7)	31773	(11.9)
Thiazides	2566	(5.3)	10439	(4.8)	13005	(4.9)
**Beta blockers, n (%)**						
No	32033	(66.7)	155382	(70.9)	187415	(70.2)
Yes	16008	(33.3)	63731	(29.1)	79739	(29.8)
**Alpha blockers, n (%)**						
No	34916	(72.7)	219113	(100.0)	254029	(95.1)
Yes	13125	(27.3)	0	(0.0)	13125	(4.9)
**TURP, n (%)**						
No	45062	(93.8)	219113	(100.0)	264175	(98.9)
Yes	2979	(6.2)	0	(0.0)	2979	(1.1)

SD, Standard deviation. ANGII, Angiotensin II Inhibitors. CCI, Charlson weighted comorbidity index. TURP, Transurethral resection of prostate

During a total of 1 417 673 person-years of follow-up with a mean follow-up of 5.3 years, there were 10 418 with a hip fracture, 19 570 with any type of fracture and 46 755 with a fall requiring in-hospital care Table 2. Compared with unexposed men, current users of 5-ARI had a multivariate-adjusted HR of 0.96 (95% CI 0.91–1.02) for hip fracture, whereas former users had an increased HR of 1.10 (95% CI 1.01–1.19) Table 2. Similarly, when all fracture types were analysed, current users again had a modest tendency of a decreased rate of fracture with an adjusted HR of 0.94 (95% CI 0.90–0.98); former users had an increased rate of fracture with an HR of 1.10 (95% 1.04–1.17). Finally, men classified as current users had an unaltered risk of falls (HR 0.99, 95% CI 0.96–1.02) and former users had a slight increased risk of falls (HR 1.11, 1.07–1.15). These results were virtually unchanged if adjustment were done for previous fractures or falls.

**Table 2:** Risk of hip fractures, any fractures and falls by 5-ARI exposure.

	Number of cases	Incidence	Univariate		Multivariate^&^		Multivariate^#^	
			HR	95% CI	HR	95% CI	HR	95% CI
**Hip fractures**								
No 5-ARI	8045	(7.24)	1.00	Ref.	1.00	Ref.	1.00	Ref.
Current 5-ARI user	1536	(8.05)	0.96	(0.91–1.01)	0.96	(0.91–1.02)	0.96	(0.91–1.02)
Former 5-ARI user	837	(7.31)	1.08	(1.01–1.16)	1.10	(1.01–1.19)	1.10	(1.01–1.19)
**Any fractures**								
No 5-ARI	15109	(13.79)	1.00	Ref.	1.00	Ref.	1.00	Ref.
Current 5-ARI user	2786	(14.81)	0.96	(0.92–1.00)	0.94	(0.90–0.98)	0.93	(0.89–0.97)
Former 5-ARI user	1675	(14.88)	1.14	(1.09–1.20)	1.10	(1.04–1.17)	1.10	(1.04–1.17)
**Falls**								
No 5-ARI	35819	(34.35)	1.00	Ref.	1.00	Ref.	1.00	Ref.
Current 5-ARI user	6865	(38.70)	1.04	(1.01–1.06)	0.99	(0.96–1.02)	0.98	(0.96–1.01)
Former 5-ARI user	4071	(38.82)	1.18	(1.14–1.22)	1.11	(1.07–1.15)	1.11	(1.07–1.15)

^&^Adjusted for previous use of Alpha blockers (Yes/No), Calcium antagonist (Yes/No), Angiotensin II antagonist (Yes/No)

Beta blockers (Yes/No), ACE inhibitors (Yes/No), Lipid-modifying agents (Yes/No), Diabetes mellitus treatment (Insulin/tablets/No), Diuretics (Thiazides/Potassium-sparing agents/High ceiling diuretics/Combination diuretics/No), Previous TURP (Yes/No), Educational level (Low/medium/High), CCI (0,1,2,3+), Civil status (Single, not single)

^#^Adjusted for ^&^ and previous fractures 0–1 years before start of follow up (Yes/No), previous fractures 1–5 years before start of follow up (Yes/No), previous hospitalisation due to fall 0–1 years before start of follow up (Yes/No), previous hospitalisation due to fall 1–5 years before start of follow up (Yes/No).

**Fig 1:**
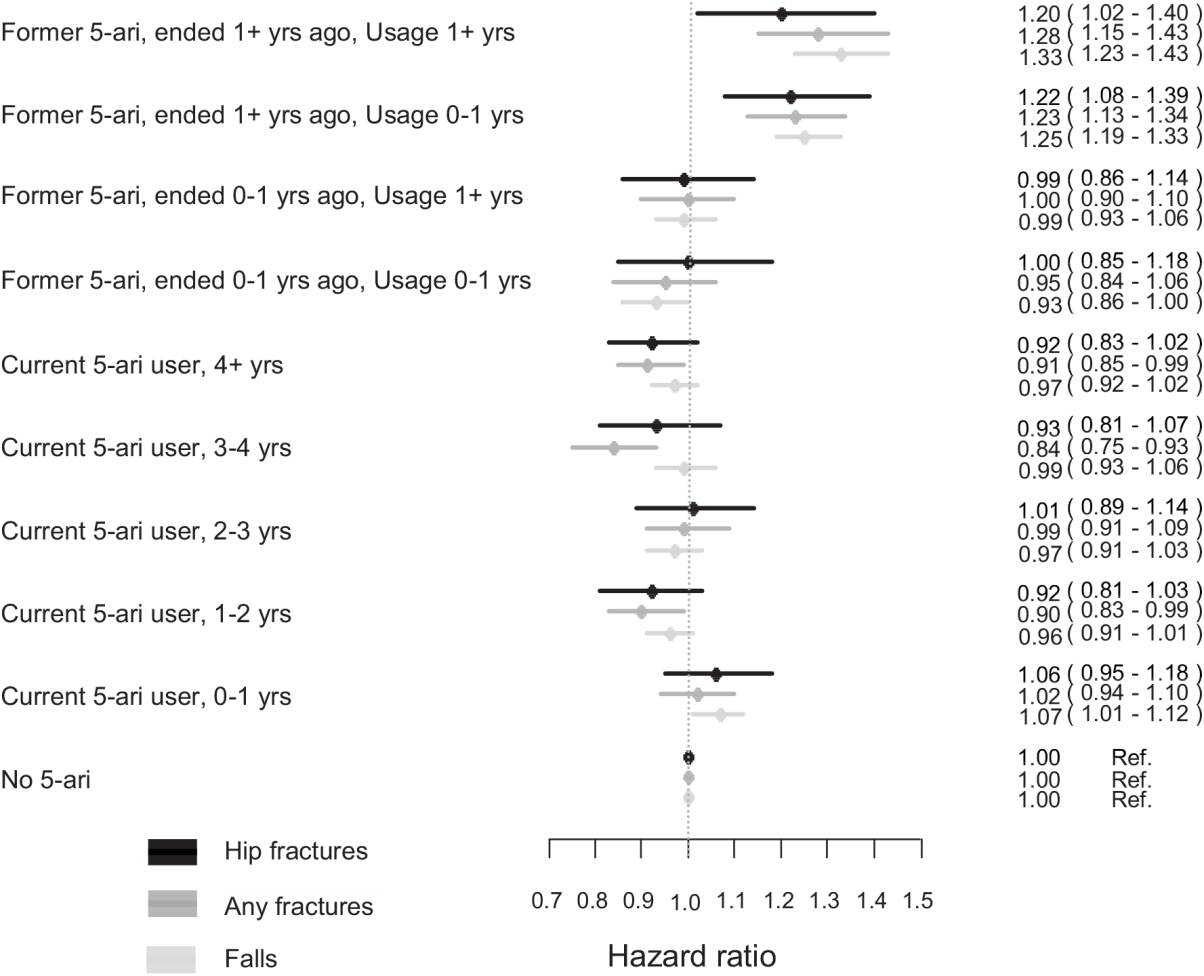
Risk of hip fractures, any fractures and falls by exposure to 5-ARI for the full cohort of former users separated according to duration of use and time since last use and for current users into four categories divided into duration of current use (<1, 1–2, 2–3, 3–4 and >4 years).

Although men with current usage for more than 4 years conferred an HR of 0.92 (95% CI 0.83–1.02), there was no clear trend in risk of hip fracture associated with prolonged 5-ARI exposure (Fig 1). Similarly, no clear trend could be established in the rates of any type of fracture by duration of use, although current use for 3–4 years led to an HR of 0.84 (95% CI 0.75–0.93) and current use for more than 4 years an HR of 0.91 (95% CI 0.85–0.99). Duration of current use was not related to the rates of falling.

Compared with never users of 5-ARI, men with former use that had ended their use within the last year had a risk close to unity for any of the outcomes. However, former use that had ended more than 1 year ago was, irrespective of duration of use, associated with a 20–30% higher rate of hip fracture, of any type of fracture and of falls.

**Fig 2:**
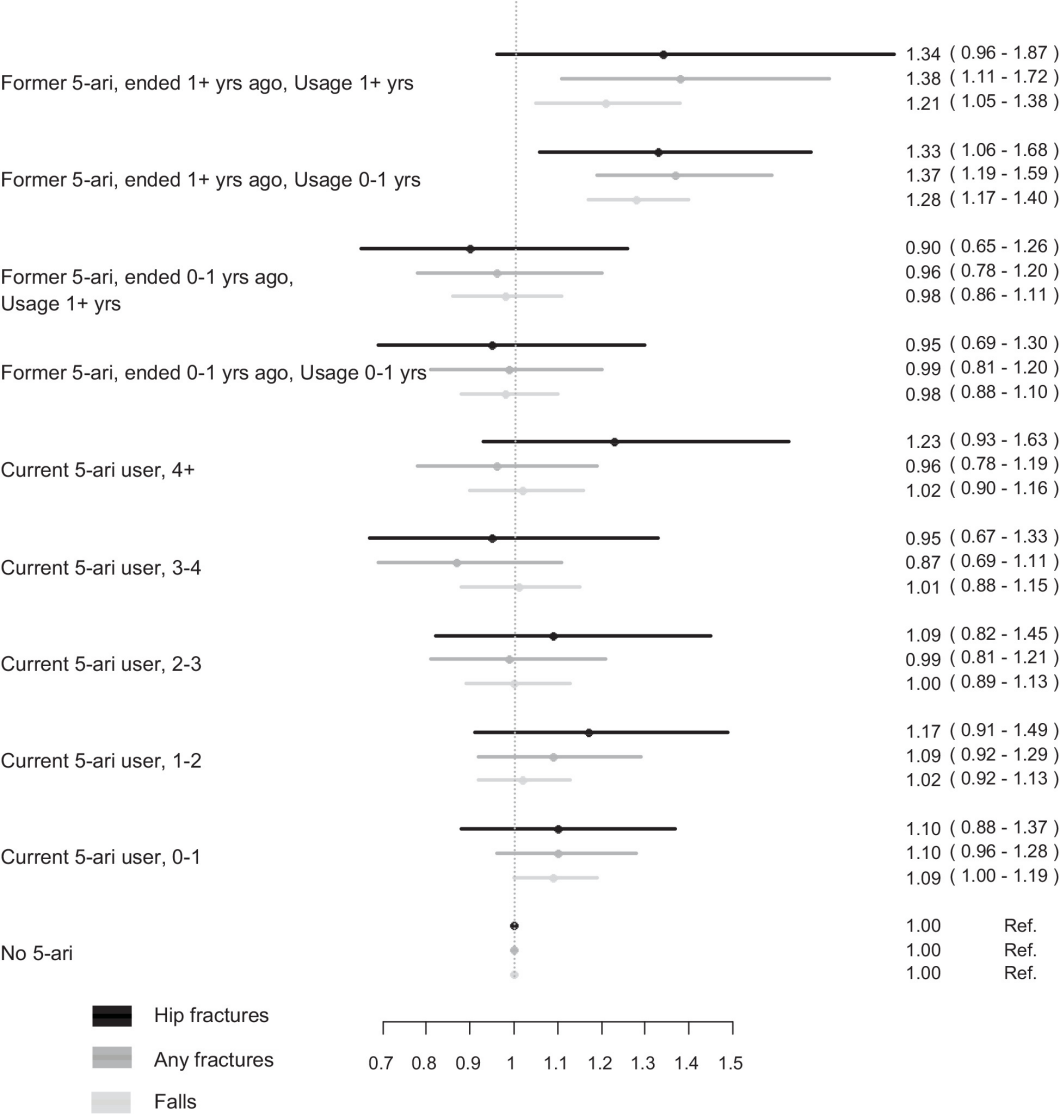
Risk of hip fractures, any fractures and falls by exposure to 5-ARI for men with a known start date for former users separated according to duration of use and time since last use and for current users into four categories divided into duration of current use (<1, 1–2, 2–3, 3–4 and >4 years).

Analysis restricted to men with a known start date of 5-ARI showed that these men (mean age 67 years) were 5 years younger than the full cohort. Expectedly, the proportion of men without comorbidity was higher than the original intact data set but the consumption of medication associated with the metabolic syndrome was reasonably the same Table 3. Although the numbers of events were lower, the results were comparable with the complete case analysis but with a slightly higher risk of fracture for current users Table 4. We could not identify any clear trends of hip fracture, any fracture or fall with prolonged 5-ARI exposure (Fig 2). For the sensitivity analysis, we found similar risk patterns between finasteride and dutasteride Table 5.

**Table 3:** Baseline characteristics of the study base for 5 alpha reductase inhibitors (5-ARI) exposed with known start date and selected non-exposed men.

	5-ARI (n = 19 614)		Non-exposed (n = 84 596)		All (n = 104 210)	
**Age, Mean (SD)**	67.1	(11.0)	67.0	(11.3)	67.0	(11.2)
**Education level, n (%)**						
High	4903	(25.0)	17923	(21.2)	22826	(21.9)
Middle	7575	(38.6)	32361	(38.3)	39936	(38.3)
Low/Missing	7136	(36.4)	34312	(40.6)	41448	(39.8)
**CCI, n (%)**						
0	13046	(66.5)	58988	(69.7)	72034	(69.1)
1	3380	(17.2)	13240	(15.7)	16620	(15.9)
2	1716	(8.7)	6949	(8.2)	8665	(8.3)
3+	1472	(7.5)	5419	(6.4)	6891	(6.6)
**Calcium Antagonist, n (%)**						
No	16334	(83.3)	72766	(86.0)	89100	(85.5)
Yes	3280	(16.7)	11830	(14.0)	15110	(14.5)
**ACE inhibitors, n (%)**						
No	15961	(81.4)	70532	(83.4)	86493	(83.0)
Yes	3653	(18.6)	14064	(16.6)	17717	(17.0)
**ANGII, n (%)**						
No	17070	(87.0)	76054	(89.9)	93124	(89.4)
Yes	2544	(13.0)	8542	(10.1)	11086	(10.6)
**Statins, n (%)**						
No	14600	(74.4)	65876	(77.9)	80476	(77.2)
Yes	5014	(25.6)	18720	(22.1)	23734	(22.8)
**Diabetes, n (%)**						
No diabetes	17677	(90.1)	76324	(90.2)	94001	(90.2)
Insulin	500	(2.5)	2559	(3.0)	3059	(2.9)
Oral drugs	1437	(7.3)	5713	(6.8)	7150	(6.9)
**Diuretics, n (%)**						
No diuretics	15815	(80.6)	68946	(81.5)	84761	(81.3)
Potassium-sparing diuretics	315	(1.6)	1340	(1.6)	1655	(1.6)
Combined diuretics	650	(3.3)	2716	(3.2)	3366	(3.2)
Loop diuretics	1747	(8.9)	7358	(8.7)	9105	(8.7)
Thiazides	1087	(5.5)	4236	(5.0)	5323	(5.1)
**Beta blockers, n (%)**						
No	13685	(69.8)	61701	(72.9)	75386	(72.3)
Yes	5929	(30.2)	22895	(27.1)	28824	(27.7)
**Alpha blockers, n (%)**						
No	11906	(60.7)	84596	(100.0)	96502	(92.6)
Yes	7708	(39.3)	0	(0.0)	7708	(7.4)
**TURP, n (%)**						
No	18081	(92.2)	84596	(100.0)	102677	(98.5)
Yes	1533	(7.8)	0	(0.0)	1533	(1.5)

SD, Standard deviation. ANGII, Angiotensin II Inhibitors. CCI, Charlson weighted comorbidity index. TURP, Transurethral resection of prostate

**Table 4:** Risk of hip fractures, any fractures and falls by 5-ARI exposure with a known start date.

	Number of cases	Incidence	Univariate		Multivariate^&^		Multivariate^#^	
			HR	95% CI	HR	95% CI	HR	95% CI
**Hip fracture**								
No 5-ARI	1698	(4.11)	1.00	Ref.	1.00	Ref.	1.00	Ref.
Current 5-ARI user	314	(5.22)	1.06	(0.94–1.20)	1.10	(0.96–1.27)	1.09	(0.94–1.26)
Former 5-ARI user	224	(4.35)	1.07	(0.93–1.23)	1.13	(0.95–1.35)	1.12	(0.94–1.33)
**Any fracture**								
No 5-ARI	3839	(9.40)	1.00	Ref.	1.00	Ref.	1.00	Ref.
Current 5-ARI user	645	(10.85)	1.02	(0.94–1.11)	1.02	(0.93–1.13)	1.01	(0.92–1.11)
Former 5-ARI user	550	(10.85)	1.17	(1.07–1.27)	1.18	(1.06–1.32)	1.17	(1.04–1.31)
**Falls**								
No 5-ari	10667	(27.25)	1.00	Ref.	1.00	Ref.	1.00	Ref.
Current 5-ARI user	1841	(32.44)	1.10	(1.05–1.16)	1.04	(0.98–1.10)	1.02	(0.96–1.08)
Former 5-ARI user	1548	(32.42)	1.20	(1.14–1.27)	1.12	(1.05–1.20)	1.11	(1.04–1.19)

^&^Adjusted for previous use of Alpha blockers (Yes/No), Calcium antagonist (Yes/No), Angiotensin II antagonist (Yes/No)

Beta blockers (Yes/No), ACE inhibitors (Yes/No), Lipid-modifying agents (Yes/No), Diabetes mellitus treatment (Insulin/Peroral/No), Diuretics (Thiazides/Potassium-sparing agents/High ceiling diuretics/Combination diuretics/No), Previous TURP (Yes/No), Educational level (Low/medium/High), CCI (0,1,2,3+), Civil status (Single, not single)

^#^Adjusted for ^&^ and previous fractures 0–1 years before start of follow-up (Yes/No), previous fractures 1–5 years before start of follow-up (Yes/No), previous hospitalisation due to fall 0–1 years before start of follow-up (Yes/No), previous hospitalisation due to fall 1–5 years before start of follow-up (Yes/No).

**Table 5:** Sensitivity analysis when hip fracture, any fracture and falls are divided in men exposed to finasteride or dutasteride, shown as a multivariate adjusted hazard ratio (HR) with 95% confidence intervals (CI).

Finasteride	Number of cases	Incidence	Multivariate	
**Hip fracture**			HR	95% CI
No 5-ARI	8026	(7.24)	1.00	Ref.
Current 5-ARI user	1296	(8.67)	0.97	(0.91–1.03)
Former 5-ARI user	637	(7.80)	1.12	(1.03–1.23)
**Any fracture**				
No 5-ARI	15069	(13.79)	1.00	Ref.
Current 5-ARI user	2312	(15.70)	0.94	(0.89–0.98)
Former 5-ARI user	1246	(15.52)	1.11	(1.04–1.18)
**Falls**				
No 5-ARI	35713	(34.36)	1.00	Ref.
Current 5-ARI user	5577	(40.23)	0.98	(0.95–1.01)
Former 5-ARI user	2942	(39.32)	1.10	(1.05–1.15)
**Dutasteride**				
**Hip fracture**				
No 5-ARI	7828	(7.34)	1.00	Ref.
Current 5-ARI user	176	(6.14)	0.98	(0.84–1.14)
Former 5-ARI user	164	(5.84)	0.97	(0.82–1.15)
**Any fracture**				
No 5-ARI	14639	(13.92)	1.00	Ref.
Current 5-ARI user	342	(12.05)	0.92	(0.82–1.03)
Former 5-ARI user	356	(12.91)	0.99	(0.88–1.11)
**Falls**				
No 5-ARI	34510	(34.50)	1.00	Ref.
Current 5-ARI user	896	(32.82)	0.93	(0.87–1.00)
Former 5-ARI user	943	(36.52)	1.03	(0.95–1.11)

## Discussion

Men with BPH and long-term current treatment with 5-ARI had no clear trend in the risk of hip fracture, any fracture or falling. Discontinuation of treatment–at least after a period of non-use–is related to higher rates of both fractures and falls.

The concerns about men’s bone health originates from the fairly broad effect that 5-ARI is known to have on several tissues. Two isozymes of 5-α reductase have been identified in humans (type I and type II). Type I is primarily in the liver but can also appear in bone and cartilage, whereas type II is mainly found in the male reproductive tissue [52–54]. The inhibition of 5-α reductase type II, caused by finasteride, lowers the intraprostatic levels of DHT. Because the primary androgen in prostate is DHT, this androgen plays an active role in BPH treatment [55]. With treatment, the circulating serum concentrations of DHT decrease by 70–85% [9, 10]. Dutasteride inhibits both 5-α reductase type I and type II, causing a decreased conversion of endogenous testosterone to DHT that leads to a 93–95% decrease in serum DHT concentrations [9, 30]. This decline in serum DHT is followed by a marked rise (25%) in serum testosterone but only 5% of serum testosterone produced in men undergo 5-α reduction to form the more potent androgen DHT [9, 56]. There is some evidence that human osteoblast-like cells express predominantly 5-α reductase type I and it is possible that local DHT production may play a role in human bone homeostasis [52]. This possibility is in part supported by findings that men with osteoporosis measured as low calcaneal BMD have significantly lower levels of DHT than their controls. Testosterone levels, on the other hand, were the same for both groups [17]. However, other aspects could also be found; for instance, men with 5 α-reductase-2 deficiency syndrome and normal testosterone levels have normal BMD [57]. Men with normal BMD but with low testosterone (less than 12 nmol/l) were treated during a 36-month period with either testosterone and finasteride, or testosterone with placebo, or placebo for testosterone and placebo for finasteride. BMD increased equally and significantly for both groups on testosterone and was unchanged for men on placebo [58]. One possible explanation for these findings is that in tissues with low 5-α reductase activity, such as muscle and bone, intratissue DHT concentrations are very low relative to testosterone and can be discontinued and the androgen effect is maintained by testosterone [59].

In our study men who discontinued treatment of 5-ARI more than 1 year ago had an increased risk of falls, as well as of hip or any type of fractures. We have no information on why the men terminated their 5-ARI medication. Theoretically, it could both be mediated by increased frailty rendering permanent catheter treatment or a perceived lack of effect of the medicine. If the latter were the case, this could lead to increased micturition problems and nocturia is indeed a risk factor for hip fracture [60–62]. The fact that it took 1 year before these men increased their risk of fall and fractures might be explained by the slow progress in prostate volume after discontinuation of 5-ARI therapy, making it appear as a delayed effect.

From The Proscar long-term efficacy and safety study (PLESS), the relative risk (RR) of an osteoporotic (hip, wrist and vertebrae) fracture in the entire cohort was 0.95 (95% CI 0.45–2.01, p = 0.89). There was also a tendency of an increased risk of any fracture (RR 1.33; 95% CI 0.92–1.91; p = 0.13) [32]. The risk of falling was not studied.

Epidemiological attempts to study the relation between 5-ARI use and hip fracture risk have been few. In a case-control study from the USA, the use of 5-ARI was compared in 7076 hip fracture cases and a similar number of controls. Only 1.8% of the participants were exposed to finasteride, corresponding to 108 cases and 141 controls [34]. The crude odds ratio was 0.77, 95% CI 0.59–1.00, p = 0.04, but the estimate was not attenuated after multivariable adjustment, which, however, did not include body mass index or markers of the metabolic syndrome. No trend was observed with duration of 5-ARI use. Moreover, no association between a diagnosis of BPH or BPH surgery and the odds of hip fractures was found. In a Danish case-control study 9719 males with any fracture were included and age-matched with 29 156 controls. Use of 5-ARI was present among 6.8 of the cases and 6.2% of the controls. No altered risk of fractures for men exposed to 5-ARI was observed [35]. Similarly, in a hip fracture case-control study performed in the UK few had been under 5-ARI treatment. Thus, only 20 (0.4%) of the 4571 cases and 10 (0.2%) of the 4571 controls had used finasteride, rendering a low precision in the risk estimates. In neither of these studies was the risk of falling studied.

The present study has limitations that merit discussion. First, we used an observational design and the selection process as to why the men initiated and ended therapy is difficult to capture using register information. Comorbidity information was considered in the analysis but the possibility of residual confounding cannot be excluded. Second, our follow-up period was short to intermediate with a maximum follow-up of 6 years. Third, body stature was not measured and components of the metabolic syndrome were not actually measured. Instead, we used specific medications as surrogate measures for metabolic syndrome.

Strengths of this study are the nationwide and therefore strictly population-based approach and the cohort design with time-dependent exposure analysis. The use of the unique personal identity number (Swedish: personnummer) that is given to all Swedish residents provided complete linkage between registers, with the consequence of virtually no problem caused by loss of participants. In addition, we were able to adjust our results for existing comorbidities, medication use and education level as one aspect of socioeconomic status.

## Conclusions

We conclude that 5-ARI is safe for men’s bone health, primarily because there is no change in the risk pattern for fractures and falls during periods of use. After discontinuation of 5-ARI, there is a modest increase in the rate of fractures and falls. However, the underlying cause of these associations cannot be determined by our study design.
